# Knowledge of the coronavirus disease 2019 (COVID-19) and sleep problems among a selected sample of psychiatric hospital staff in Nigeria: a cross-sectional study

**DOI:** 10.11604/pamj.2021.40.39.25357

**Published:** 2021-09-15

**Authors:** Kehinde Oyeyemi Oderinde, Oluyemi Oluwatosin Akanni, Anthony Olashore

**Affiliations:** 1 Department of Mental Health, University of Benin Teaching Hospital, Benin City, Nigeria,; 2 Forensic Unit, Federal Neuro-Psychiatric Hospital, Benin City, Nigeria,; 3 Department of Psychiatry, University of Botswana, Gaborone, Botswana

**Keywords:** Knowledge, COVID-19, sleep problem, Nigerian neuropsychiatric hospital

## Abstract

**Introduction:**

as the coronavirus disease 2019 (COVID-19) spreads, sleep problems are expected to increase among healthcare workers. Therefore, we aimed to assess the knowledge of COVID-19, sleep problem and identify sociodemographic factors associated with sleep problems among healthcare workers in a Nigerian neuropsychiatric hospital.

**Methods:**

a cross-sectional study was conducted among 200 healthcare workers in a neuropsychiatric hospital using self-administered questionnaires to assess knowledge of COVID-19, sleep problem, social support, and sociodemographic factors that affect sleep. Chi-square test and Spearman's correlation were applied to assess the association between sociodemographic factors and sleep problems.

**Results:**

about 23.9% of the healthcare workers reported having a sleep problem. However, there was no association of sleep problems with any sociodemographic factors except age (r=0.26) and social support (r=-0.18).

**Conclusion:**

the study offered insight into the occurrence of sleep problems among healthcare workers and suggested a guide for planning interventions targeted at improving the psychological well-being of healthcare workers in the face of current global pandemics.

## Introduction

Sleep is a naturally re-occurring physiological phenomenon that is essential for rest, repair, learning, and development [1]. When it is disrupted, it could have both physical and psychological impact on the individual and the impairment of functioning at work [2]. Good sleep quality has been described as a total sleep time of 85% or more, commencing sleep not later than half an hour while attempting sleep, waking up not more than once during the night and being able to recommence sleep within 15 minutes of an initial awakening across all age groups [3]. Although sleep is expected to improve during the coronavirus disease 2019 (COVID-19) because people are forced to stay at home, this may not be true for health workers who are essential service providers because they are expected to carry on with work [4]. Before the COVID-19 pandemic, sleep problems have been recorded to be high among health workers because of multiple reasons often related to work such as long and busy hours of work, lack of support with increasing expectations, and exposure to a traumatic event [5].

Sleep problems may thus be worse among health workers in low and middle-income countries like Nigeria, who are overlaboured by work schedule [1]. Previously, some findings have been documented in Nigeria among health workers about their sleep problems [1,6]. This may be further compounded during the COVID-19 pandemic, because of the anxiety and other changes relating to the spread and prevention of the virus [7]. As at the time of data collection for this study, no study (to the best of knowledge of the investigators) existed that investigated the impact of the COVID-19 pandemic on the sleep quality of healthcare workers, therefore describing the sleep problems among this group in unprecedented times like this is undoubtedly valuable. Given the statement above and in addition to surveying knowledge of the nature, cause, treatment, and prognosis of COVID-19, we decided to assess the knowledge of COVID-19, sleep problem, identify sociodemographic factors associated with sleep problems and its relationship with social support among healthcare workers in a Nigerian neuropsychiatric hospital. The result of this study will ultimately help determine the relevance of social support and offer recommendations to this group where necessary.

## Methods

**Study design and location:** the study design was a cross-sectional descriptive one. It was conducted at the federal neuropsychiatric hospital (FNPH) in Benin-City, Edo State, South-south Nigeria. The hospital is a 270-bed facility which provides in-patient and out-patient care, as well as emergency services to mentally ill persons primarily across the region (a geographical catchment area of six states) of Nigeria. The location operates at two sites: the old site is centrally located in the city; and the new (permanent) site, located on the outskirt of the city. The sample size was calculated using single population proportion formula 21. Data was collected over one-month period between May 2020 to June 2020 using self-administered questionnaires to assess knowledge of COVID-19, sleep problem, social support, and sociodemographic factors that affect sleep.

**Eligibility criteria:** healthcare workers who were bonafide staff of the hospital, aged 18 years and above, have worked in the hospital for a minimum of one year prior to the outbreak of COVID-19 in Nigeria, reported no history of mental illness and willing to participate were included in the study. Healthcare workers who did not meet any of the above criteria were excluded from the study.

**Study population:** the study population consisted of the staff with close contact with patients working in the health facility, consultant psychiatrists, resident doctors in psychiatry, medical officers, psychiatric nurses, pharmacists/technicians, laboratory scientists/technicians, clinical psychologists, social workers, occupational therapists, health attendants, and others.

**Sampling technique:** the staff of the hospital who were present in the course of questionnaire distribution were randomly sampled. All the consenting personnel listed above that were available during the study period were recruited.

### Instruments: the following was used for the study

**Socio-demographic data collection sheet:** specifically designed to inquire about the sociodemographic characteristics of the staff, such as gender, age, marital status, education level, profession, and religion.

**Knowledge about COVID-19:** seven questions were self-designed to capture the knowledge about nature, cause, risk, treatment, fatality, prevention, and prognosis of the coronavirus disease.

**Sleep problems:** this was enquired as a single question: “do you have difficulty falling asleep, staying asleep or waking up early” and a Likert option of responses were provided, such as never, sometimes, half the time, frequently and always; to enable scoring. Higher scores are indicative of greater sleep problems.

**The Oslo 3-item Social Support Scale (OSS-3):** the OSS-3 provides a brief measure of social support [8]. It covers different fields of social support by measuring the number of people the respondent feels close to, the interest and concern showed by others and the ease of obtaining practical help from others [9]. Its structure and reliability (cronbach´s alpha) have been documented in Nigeria (0.50) to be acceptably low; nonetheless, its brevity, clarity, and the availability of normative data are strong considerations for use in this study [10]. The higher the scores, the stronger the support.

**Ethical consideration:** approval for the study was obtained from the Research and Ethics Committee of Federal Neuropsychiatric Hospital, Benin City, Nigeria, and written informed consent from the participants to qualify for recruitment. The ethical approval obtained was PH/A. 864/vol. XV/129. the study questionnaires were administered within one month to the study population after obtaining consent.

**Statistical analysis:** data collected were analyzed using the statistical package for social sciences (SPSS) version 22. Descriptive statistics were used, such as frequencies and median. A normality test was performed, which revealed that the sleep problem score was not normally distributed, hence a non-parametric test was adopted in bivariate analysis. Chi-square test was used to test the association between sleep problem and categorical variables (gender, marital status, religion, profession) while Spearman´s correlation was applied between sleep problem and continuous variables (age, social support). All the variables except profession were dichotomized for easier comparison with previous studies and to enable enough participants for analysis e.g. the educational status was categorized into at most secondary level of education and at least tertiary education. The profession was categorized into doctors, nurses, and others (psychologists, social workers, and occupational therapists). The level of significance was set at a p-value of less than 0.05.

## Results

Table 1 shows that two hundred questionnaires were analyzed, and the participants sampled were 40.0 years of median age, 71 males (35.5%), and 129 females (65.5%). The nursing staff (51.6%) formed the highest percentage of the workforce, while most of the workers were Christians (93.0%) and married (79.4%). Concerning sleep, 150 (76.1%) workers reported ‘never´ having any difficulty, while 19.8% admitted having problems 'sometimes'. No one admitted to having sleep difficulties ‘frequently´; however, 2% stated they had sleep problems ‘half the time´ and ‘always'. Hence, the total of those who reported having problems (sometimes-always) with their sleep was 47 (23.9%) (Table 1).

**Table 1:** sociodemographic characteristics of participants

Variables	Characteristics	No of participants (%)
**Gender**	Male	79 (35.5%)
	Female	121 (64.5%)
Educational qualification*	Primary	1 (0.5%)
	Secondary	21 (10.9%)
	Tertiary	115 (59.9%)
	Post tertiary	55 (28.6%)
Marital status*	Single	37 (18.6%)
	Married	158 (79.4%)
	Separated/widowed	3 (1.5%)
	Widowed	1 (0.5%)
Religion*	Christianity	185 (93.0%)
	Islam	12 (6.0%)
	African traditional religion	2 (1.0%)
Profession*	Doctor	15 (8.0%)
	Nurse	97 (51.6%)
	Pharmacist	9 (4.8%)
	Lab scientist	9 (4.8%)
	Health attendant	20 (10.6%)
	Social worker	15 (8.0%)
	Others^	23 (12.2%)
Sleep problem*	Absent	150 (76.1%)
	Present	47 (23.9%)

Median age is 40 years * Figure did not add up to 200 because of missing data ^ Others are clinical psychologist, occupational therapist, medical record officer, etc

Concerning their knowledge about COVID-19, Table 2 shows that 30.2% knew the infection is airborne, while just over half (55.9%) were correct in their estimation of the fatality rate of the infection. Most of the participants (87.5%) felt the risk of contracting the illness is high or very high, and similarly, a great proportion of persons (76.8%) opined that the illness could be prevented. Also, most of the respondents (94.5%) indicated that the cause of the infection is viral, treatment is medical (95.3%), and recovery is possible (90.9%) (Table 2). Table 3 shows that there was no association of sleep problem with any of the sociodemographic factors studied, the exception to this where age (r=0.26) and social support (r=-0.18) as shown in Table 4. The significant relationships imply that as age increases, sleep problem increases while as social support diminishes, sleep problem increases.

**Table 2:** COVID-19 knowledge of participants

Variable	Characteristics	No of participants (%)
Airborne infection*	Yes	57 (30.2%)
	Not certain	53 (28.0%)
	No	79 (41.8%)
Death rate of infected people*	1-10%	95 (55.9%)
	11-20%	9 (12.4%)
	21-30%	19 (11.2%)
	31-40%	21 (12.4%0
	41-50%	26 (15.3%)
Perceived risk of contraction*	Very low	2 (1.0%)
	Low	5 (2.6%)
	Moderate	17(8.9%)
	High	55 (28.6%)
	Very high	113 (58.9%)
Can COVID-19 be prevented*	Yes	152 (76.8%)
	Not certain	43 (21.7%)
	No	3 (1.5%)
Cause of COVID-19*	God's punishment	10 (5.0%)
	Evil spirit	1(0.5%)
	Viral disease	188 (94.5%)
Treatment for COVID-19*	Spiritual	9 (4.7%)
	Medical	181 (95.3%)
Recovery from COVID-19*	Yes	170 (90.9%)
	Not certain	16 (8.6%)
	No	1 (0.5%)

* Figure did not add up to 200 because of missing data

**Table 3:** association between sleep problem and participants' characteristics

Variables		Sleep problem		p-value
		Absent (%)	Present (%)	
**Gender**	Male	49 (35.0%)	17 (37.8%)	0.74
	Female	91 (65.0%)	28 (62.2%)	
**Marital status**	Married	118 (79.2%)	38 (80.9%)	0.81
	Others	31 (20.8%)	9 (19.1%)	
**Educational status**	Sec and below	17 (11.6%)	5 (11.9%)	0.95
	Ter and above	130 (88.4%)	37 (88.1%)	
Religion*	Christianity	137 (91.9%)	45 (95.7%)	0.52
	Others	12 (8.1%)	2 (4.3%)	
Profession*	Doctor	14 (10.1%)	1 (2.2%)	0.14
	Nurse	69 (49.6%)	27 (58.7%)	
	Others	56 (40.3%)	18 (39.1%)	

* Fisher's exact test/Yate correction

**Table 4:** spearman’s inter-correlation of variables

Measures	1	2	3
**1. Sleep problem**	1		
**2. Social support**	-0.18*	1	
**3. Age**	0.26**	0.03	1

Correlation is significant at the 0.05 level (2-tailed) Correlation is significant at the 0.01 level (2-tailed)

## Discussion

This hospital-based cross-sectional study examined the knowledge of COVID-19 and sleep problems among a selected sample of healthcare workers in a neuropsychiatric hospital in the South-south region of Nigeria. This survey outcome can help with making health recommendations and formulating right precautionary measures, which will invariably boost the psychological and physical health of the healthcare workers during and after the pandemic [11]. The healthcare workers that participated included medical doctors, nurses, pharmacists, medical laboratory scientists, clinical psychologists, social workers, occupational therapists, medical record officers, and other mental health support staff. The finding in our study showed that the vast majority of the participants had good knowledge of COVID-19, this is similar to other previous studies about COVID-19 in Vietnam and China, where a large proportion of healthcare workers were aware and knowledgeable about the pandemic [12,13]. This might be due to a general consequence of the heightened awareness of COVID-19 among healthcare workers who are likely to be more educated about the pandemic. Understanding the knowledge of COVID-19 among healthcare workers is essential to bring forth a sustained change in behaviors and to improve such practices when designing interventions [11]. Also, more enlightenment is necessary to communicate information to the minority of the healthcare workers in this study who lack sufficient knowledge of issues related to COVID-19.

Our study also indicated that 23.9% of the respondents had a sleep problem. This rate is consistent with several studies that have reported the prevalence of sleep problems among healthcare workers, in the range of 10-30% [14,15]. However, the percentage obtained in our study was lower than that reported by Lai *et al*. [16], where they found that 34% of their participants had sleep problem and that working outside Wuhan (origin and epicenter of the epidemic in China) is associated with a lower risk of experiencing distress and sleep problem. The difference in percentage between the two studies can be attributed to the direct experience of the COVID-19 devastation by the Chinese population at the early stage of the outbreak and the Nigerian population observation of the outbreak through mass media. Sources of sleep problems among healthcare workers during the pandemic may include feelings of vulnerability, loss of control, and concerns about the health of the self and health of their immediate families [17]. It was observed in our study that social support was associated with sleep problems among healthcare workers. This is consistent with the findings of Xiao *et al*. [7], where they found that psychological well-being and sleep are affected by the level of social support for healthcare workers. This is because social support reduces anxiety levels by decreasing the perception of the threat of stressful events and the physiological and inappropriate behaviors that can result from stress [18]. The finding from this study may provide endorsement for the implementation of measures to improve the social support and network of healthcare workers during increased demands associated with the COVID-19 pandemic at this time.

Another important finding in this study was that increasing age was also significantly associated with sleep problems. This is in tandem with the findings of Patterson *et al*. [19], where they reported that some sociodemographic factors such as increasing age and level of social support were important determinants of sleep problems among healthcare workers. This is because, as people age, a higher number of medical conditions may develop, which tends to have a negative effect on sleep [19]. However, this is contrary to the findings of Ogunbode *et al*. [6] where there reported no association between age and sleep problem. This study found no association between sleep problems and some sociodemographic factors such as gender, marital status, and educational status. This agrees with the findings of Ghalichi *et al*. [20], in an Iranian study to assess sleep quality among healthcare workers. Additionally, this current study provides baseline data on the current situation of sleep problems among healthcare workers during the COVID-19 pandemic and will serve as a basis for future research. The study is limited by its cross-sectional design and the fact that some respondents could have given socially acceptable answers to some questions. The survey being a single-center study makes generalization of findings to other health facilities a limitation and the questionnaire used for the study was not validated also. However, the study results provide baseline data on the current sleep problems among healthcare workers in Nigeria during the COVID-19 pandemic and will serve as a basis for future research. Furthermore, the findings can be used for future planning to address the occupational health of healthcare workers.

## Conclusion

Most of the health workers in this study were well aware of the etiology of COVID-19 disease, treatment and recovery of individuals with COVID-19. The study also showed that approximately 24% of the healthcare workers reported having sleep problem during the pandemic. It is therefore recommended that during pandemics, preventive interventions are put in place for the overall mental and psychological being of healthcare workers.

### What is known about this topic

- Healthcare workers do experience poor sleep quality with relevant consequences on their health;
- Poor sleep quality among healthcare workers is prevalent and this has increased during the COVID-19 pandemic;
- Several variables such as educational level or work in an isolation unit appear to play a crucial role in moderating sleep disturbance.

### What this study adds

- Sleep disturbance was prevalent among the healthcare workers and sociodemographic variables increasing age and lower level of social support were associated with sleep problem among the study population.
